# Sensing Technologies for Extravasation Detection: A Review

**DOI:** 10.1021/acssensors.2c02602

**Published:** 2023-03-13

**Authors:** Ikue Hirata, Arianna Mazzotta, Pooyan Makvandi, Ilaria Cesini, Chiara Brioschi, Andrea Ferraris, Virgilio Mattoli

**Affiliations:** # Center for Materials Interfaces, Istituto Italiano di Tecnologia, 56025 Pontedera, Pisa, Italy; ‡ The Biorobotics Institute, Scuola Superiore Sant’Anna, Pontedera 56025, Italy; § IIT-Bracco Joint Lab, Istituto Italiano di Tecnologia, 16163 Genova, Italy; ∥ Bracco S.p.A., 20134 Milano, Italy

**Keywords:** Blood extravasation, infiltration, bleeding, intravenous catheters, contrast agent, drug infusion, radiofrequency sensor, ultrasound sensors, infrared sensors, thermal imaging, PIVC

## Abstract

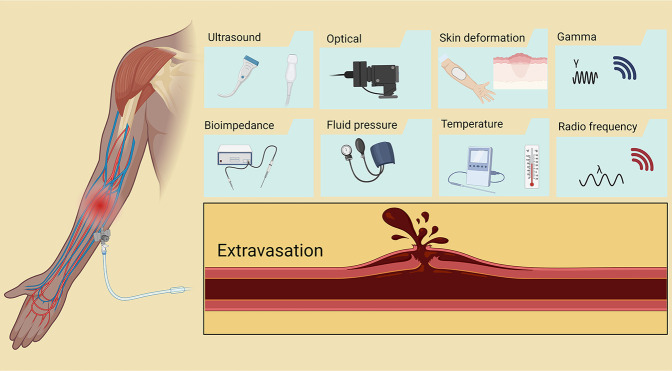

Peripheral intravenous catheters are administered for various purposes, such as blood sampling or the infusion of contrast agents and drugs. Extravasation happens when the catheter is unintentionally directed outside of the vein due to movement of the intravascular catheter, enhanced vascular permeability, or occlusion of the upstream vein. In this article, extravasation and its mechanism are discussed. Subsequently, the sensorized devices (e.g., single sensor and multimodal detection) to identify the extravasation phenomena are highlighted. In this review article, we have shed light on both physiological and engineering points of view of extravasation and its detection approaches. This review provides an overview on the most recent and relevant technologies that can help in the early detection of extravasation.

Peripheral intravenous cannulation (PIVC) consists of the insertion of an indwelling single lumen catheter into a peripheral vein by puncturing the skin.^1^ Peripheral intravenous cannulation is one of the most common invasive procedures performed in hospitals as it is required in a wide range of clinical applications. For instance, it allows the direct introduction of fluids, medications, and other therapies into the cardiovascular system so as to rapidly reach the target organs.^1,2^ Up to 70% of patients require a peripheral venous line during their hospital stay with over 1 billion IV insertions taking place in the United States annually.^3,4^ Despite the high number of patients requiring this procedure, IV insertions still require the intervention of trained and experienced physicians and nurses.^3^ Indeed, the practitioner has to insert the cannulation needle into the vein but without double puncturing it, in order to consequently slide off the needle, which is then retracted.^5,6^

The only way the practitioner has to ensure that the cannula is positioned correctly is to feel a small change in the force of insertion on the needle when the first wall of the vein is punctured and then immediately stop. Hence, this procedure is mainly based on the experience and specialized technique of the practitioner, leading to the evidence that younger or less experienced nurses have a significantly lower success rate than their senior counterparts (failure rates are in the range 9–56%).^7^ When the catheter penetrates through the second vessel wall, it could easily cause extravasation leading to an infiltration of a known vesicant or caustic agent into the surrounding tissues^8^ and causing redness, pruritus, allergic reactions or edema, as well as discomfort or pain expressed by the patients.^8−10^ Furthermore, in the case of injection of chemotherapeutic agents, the effect of the extravasation damage could not be immediate but may evolve over days or weeks.^11,12^ Among the various complications related to extravasation, some of the most serious are full thickness skin loss or muscle and tendon necrosis for which reconstructive surgery is required, resulting in a prolonged hospitalization of the patient and increased morbidity.^8,10^ Notably, extravasation injuries often occur in children and infants (incidence rate of 18–46%) and lead to serious complications also related to their growth.^13−15^

Therefore, it is of crucial importance to detect extravasation at very early stages. For example, patient’s skin swelling and deformations, changes in the color of the skin, bioimpedance, temperature, and blood flow rate represent some of the parameters that could be monitored to prevent/detect extravasation.^16−19^ Extravasation is still an unsolved issue in the medical practice. Therefore, we believe that this review, which offers an overview of the latest and most relevant devices, can aid in better understanding of what is still needed to improve IV administration procedures.

Herein, extravasation and its mechanism are highlighted. Afterward, the readers will be introduced to an overview related to the existing sensory devices developed to date to identify extravasation, then going into details by reviewing different types of technologies (i.e., both single and multimodal detection sensors), analyzed on the base of the detection method—i.e. radio frequency (RF)/microwave reflection and absorption by the tissue around the cannulation site, optical/infrared refraction/reflection, ultrasound mapping, strain/pressure monitoring, thermal mapping, and external bioimpedance measurements.

Extravasation is still an unsolved issue in the medical practice. Therefore, we believe that this review, which offers an overview of the latest and most relevant devices can aid in better understanding what is still needed to improve IV infiltration procedures.

## Extravasation Mechanism, Management, and Treatment

Annually, about 2 billion peripheral intravenous catheters are employed (e.g., for blood sampling or administration of contrast agents and drugs) worldwide.^20^

**Figure 1:**
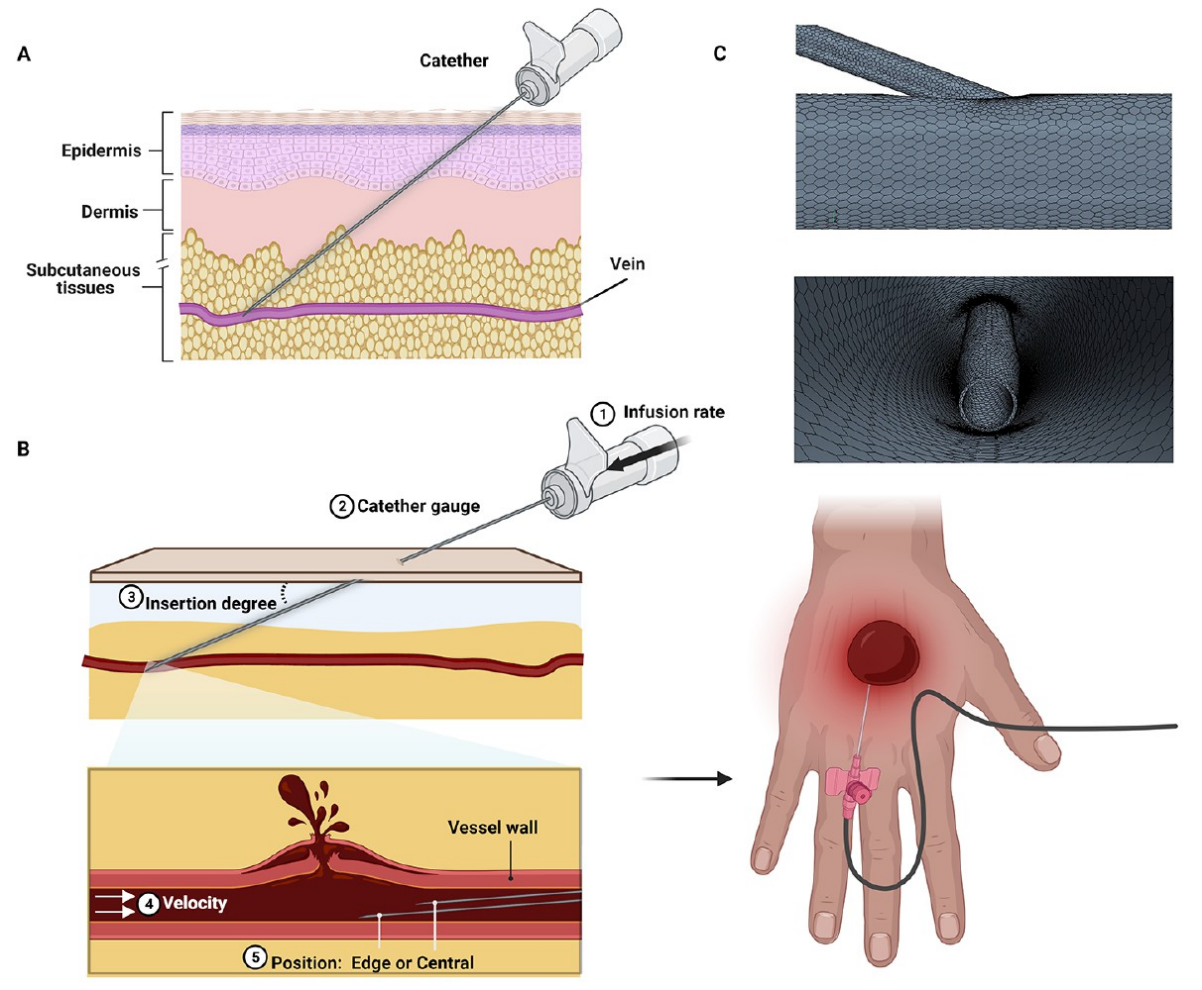
(A) Schematic illustration of skin layers and vein. (B) Diagram of parameters that affect extravasation. (C) Representative computational mesh on the 20 G, 20° insertion angle, positioned at the vessel edge from the side and internal views. Part C reprinted from ref (22) under open access license.

Skin comprises three layers including epidermis, dermis and subcutaneous tissues (Figure 1A). The catheter/cannula should pass the skin to reach and also remain in the vein in the subcutaneous tissue. Extravasation happens when the catheter is not properly inserted into the vessel or unintentionally directed outside of the vein due to movement of the patient, excessive pressure when an infusion pump is used, an enhanced vascular permeability, or occlusion of the upstream vein. It should be noted that the extravasation rate is about 40–60% for patients who require intravenous cannulation.^16^ Extravasation injury results from the leaking fluid into perivascular space or subcutaneous tissue, as well as cannula moves out of the vein, causing tissues damage. This is associated with some risks, e.g., from local irritation to severe tissue loss. The tissue reaction relies on different factors, such as the chemical nature and the amount of the leaked fluid, the site of extravasation, and the relative size, age, and condition of the patient.^21^

Patients of any age are susceptible to extravasation; however, neonates and elderly patients who possess thin and poorly supported skin and subcutaneous tissue are more susceptible to this phenomenon.^23^ For instance, infants, children, and unconscious patients are more at risk for extravasation because they are unable to complain about the pain associated with the administration site. On the other hand, elderly patients and people with low muscle mass and atrophic hypodermic tissue are more prone to intensive extravasation injuries. Besides, patients with arterial insufficiency or compromised venous drainage or lymphatic drainage can be able to endure extravasation less than people with normal circulation. Besides these groups, patients undergoing chemotherapy are susceptible to extravasation since chemotherapy may induce vascular fragility.^24−26^ It should be noted that longer duration and deeper PIVC are independent risk factors that predisposed patients to extravasation.^27^

**Figure 2:**
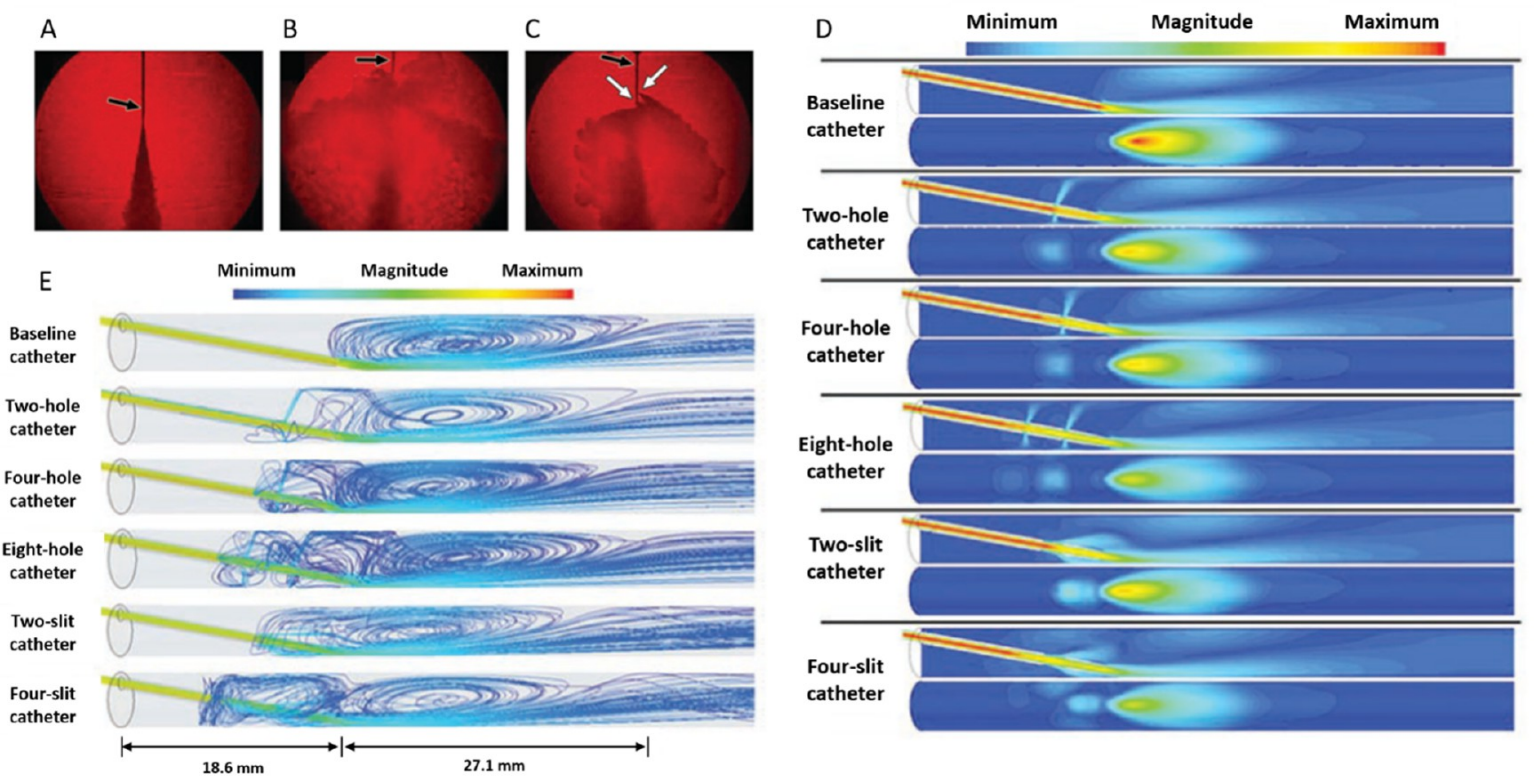
(A–C) Schlieren images of contrast jets exiting end hole only (A), four side holes (B), and four side slits (C) catheters. (D) Numerical simulations of the velocity profile (upper model) and the wall shear stresses (lower model) for multiple catheter types with the catheter tip directed at an oblique angle in a 5 mm vessel wall; (E) Fluid flow path lines for multiple catheter types in a 5 mm vessel. Reprinted with permission from ref (29). Copyright 2009 American Roentgen Ray Society.

The mechanism of extravasation injury is not fully rationalized; however, it seems that the amount of tissue damage depends on pH, osmolality, and ion dissociability, as well as direct cytotoxicity of the infusate.^21,28^ Given this, computational fluid dynamics was employed to assess typical peripheral intravenous catheters factors including infusion rate, size of the catheter (measured in gauge), insertion angle, blood velocity, and the position of needle tip on the resulting parameters (e.g., shear stress to the blood vessel wall, blood damage, particle residence time and venous stasis volumes).^22^ The results exhibited that the most significant parameter is the infusion rate of infusate from the catheter, where excessive injection rates may damage the vein wall and the blood (Figure 1B and C).^22^ Weber et al.^29^ investigated the possibility to use different designs of the catheters (e.g., catheters with two side holes, four side holes, eight side holes, two side slits, and four side slits) with respect to the more standard single end-hole one (Figure 2A–C) in a diagnostic scenario. At high flow rates, the jet of contrast medium exiting the catheter may cause vascular damage. With the introduction of four lateral holes and four lateral slits, the velocity of the jet is decreased, likely reducing the probability of damaging the vessel. From a quantitative point of view, numeric flow simulations reported reduced velocity at the level of the catheter tip and also reduced shear stresses in the case of the other configurations compared to the standard one (Figure 2D). Nevertheless, path lines of the injected fluid depicted in Figure 2E show that the flow is highly turbulent in the vicinity of the side holes or side slits for all catheters and this aspect causes increasing shear stress of the fluid on the vessel walls. These results underline that a different design of the catheter could help in minimizing the possibility of damage to the vessel due to the jet of the contrast medium.^29^

There is no consent about the best method for the treatment of extravasation.^24^ However, some approaches have been utilized. For instance, elevation of the injured limb to reduce the hydrostatic pressure in capillaries is beneficial to decline the edema. Besides, immediate warm or cold compresses on the injured site are performed for the treatment. After these steps, different ointments (e.g., silver sulfadiazine) are prescribed for the prevention of secondary infection if needed. Other treatments involve the administration of hyaluronidase which is an enzyme that breaks down connective tissue as well as dimethyl sulfoxide which is a free-radical scavenger, possessing antibacterial, anti-inflammatory, and vasodilatory effects, as far as the surgical intervention in most severe damages.^30−33^ However, the majority of plastic surgeons recommend a conservative policy for the treatment of extravasation injuries without surgery.^24^

## Extravasation Detection Technologies: Overview

**Figure 3:**
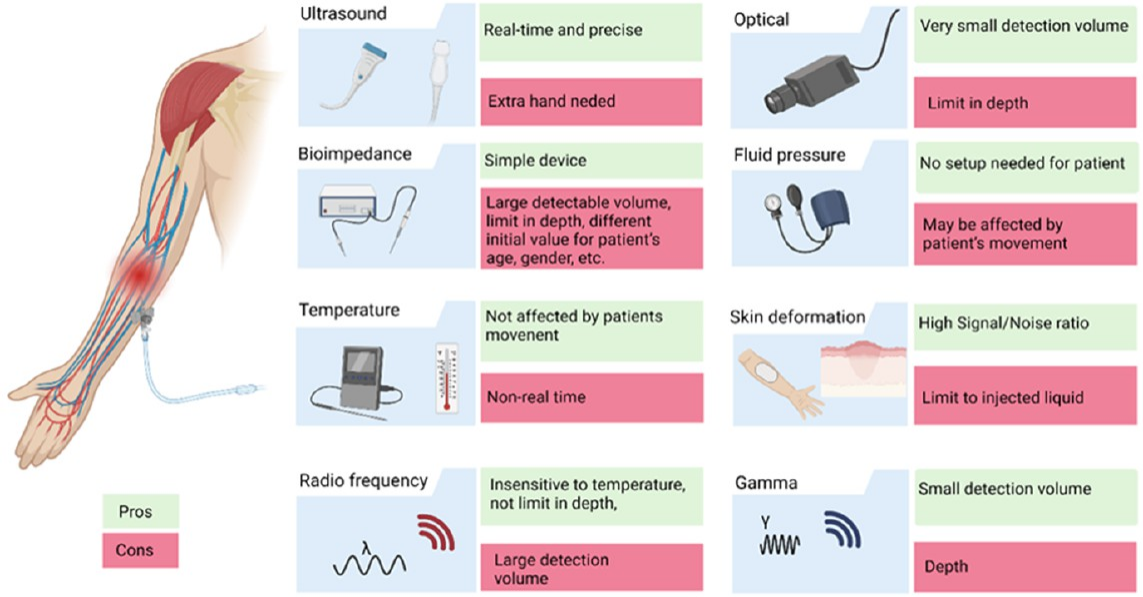
Sensor-based extravasation detection approaches along with their advantages and disadvantages.

Currently, the occurrence of the event is mostly identified by visual and tactile inspections for skin discoloration and swelling. Therefore, during the course of the injection, a major role is posed onto the clinicians and the nursing personnel to detect the extravasation event.^34−36^ To support the inspection and to anticipate the detection, several applications are proposed, by applying biophysical measurement techniques, such as bioimpedance, thermographic mapping, radiofrequency (RF) reflection/absorption, optical refraction/reflection, ultrasound mapping, fluid pressure measurement, skin deformation, gamma scintillation sensing, and the exhaled carbon dioxide sensing. These sensors are evaluated in several aspects: the device format, the portability and the ease of the sensor setup, the availability as commercial products, sensitivity, specificity, the minimum detectable volume of fluid, the position of the device. They are summarized in Table 1 and visualized in Figure 3.

**Table 1:** Summary of the Sensors Used for the Detection of the Extravasation Incidents

sensing parameter	device format	commercialized	sensitivity	specificity^b^	minimum detectable volume (ionic/nonionic liquids)	position	ref.
bioimpedance	patch	yes	100%	N.A.^a^	11.6/13.5 mL	attached to skin	(37, 38)
temperature	thermal camera, liquid crystal film	no	85%	N.A.	N.A.	external (camera), attached to skin (film)	(39−41)
RF/microwave	small solid boxes	yes	99.8%	99.97%	20 mL/N.A.	attached to skin	(42, 43)
optical	optical fiber/device	yes	93%	95%	0.1 mL/N.A.	attached to skin	(44−47)
ultrasound	ultrasonographic scope, monitor	yes	100%	100%	N.A.	close to the site	(48, 49)
fluid pressure	pressure sensor at power injector	yes	N.A.	N.A.	N.A.	external (power injector)	(50)
skin deformation	patch	no	N.A.	N.A.	2 mL/N.A.	over the site	(16, 51−54)
gamma scintillation	small solid box	yes	N.A.	N.A.	N.A.	attached to skin	(55)

a N.A.: Not applicable.

b Specificity: The ratio of nonextravasation cases among all the cases where the sensor did not detect extravasation.

In literature, multiparametric approaches are also found, such as bioimpedance and skin deformation or body temperature and skin deformation. In the following section, single- and multisensor-based extravasation detection approaches are represented, highlighting their pros and cons.

## Sensorized Devices

### Single Sensor

In recent years, the demand for biosensors has grown,^56^ showing potential use also in extravasation detection.^75^ Single sensor refers to a device targeting a single specific biophysical information to to be measured. In the following sections, sensors based on bioimpedance, ultrasonic image, radiofrequency transmission, ultrasound, fluid pressure, and skin strain are presented.

#### Impedance Sensor

**Figure 4:**
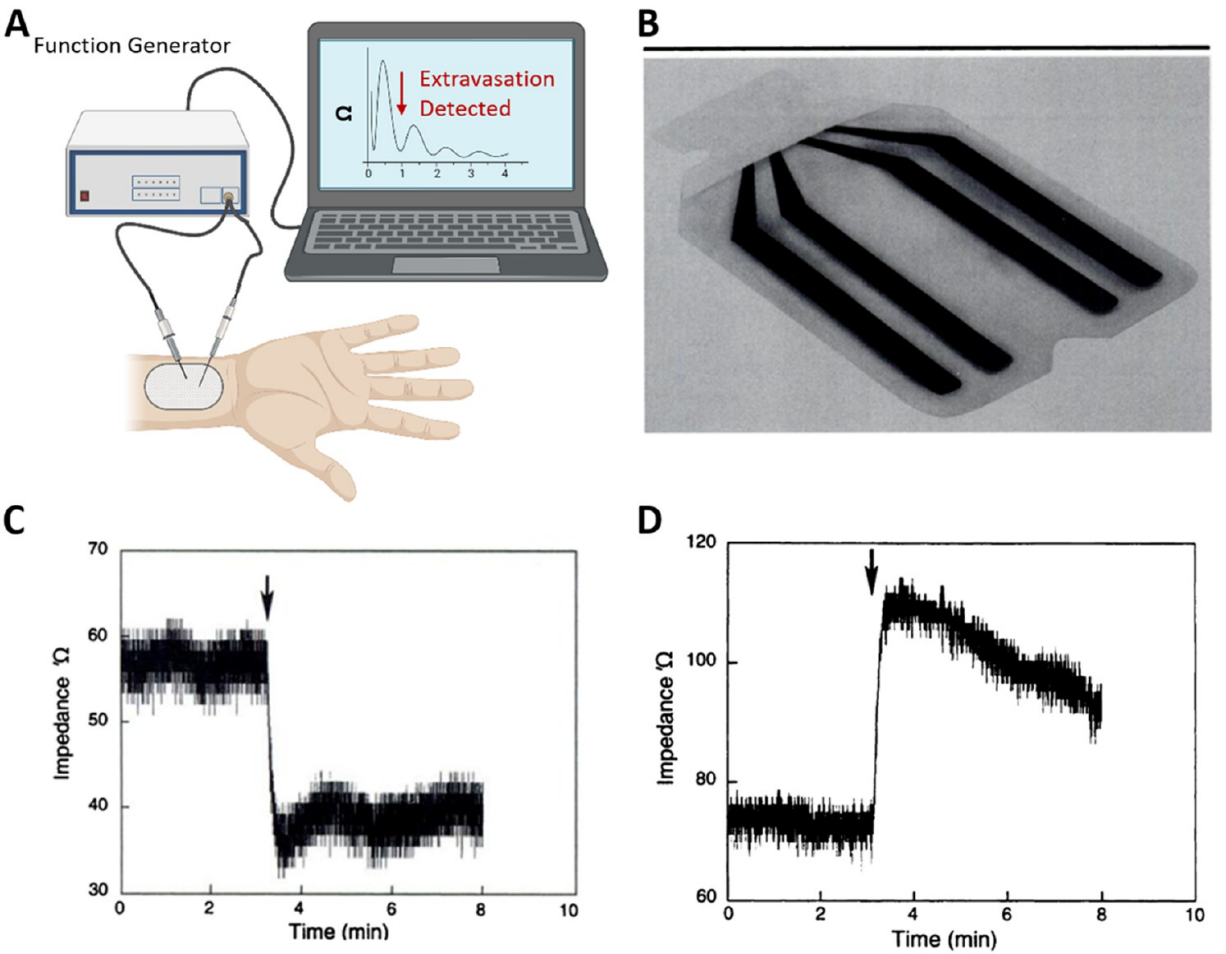
(A) A schematic of the impedance measurement system. (B) A completed sensor patch with tetrapolar impedance sensor, with the dimension of 5 × 8 cm. (C) Impedance response during the extravasation of an ionic agent on the hind leg of a dog. A sharp decrease of the impedance is observed. (D) Impedance response during the extravasation of a nonionic agent on the hind leg of a dog. A sharp increase of the impedance is observed. Reprinted with permission from ref (37). Copyright 1998 Radiological Society of North America.

Extravasation evidenced by the change of the skin surface impedance is based on the difference between the impedances of the skin and the injected fluid. A patch with multiple electrodes is placed on the location where the catheter is inserted. A weak high-frequency alternating current passes between two electrodes, and the impedance value is read between the other pair of electrodes. When the fluid accumulates at a certain location, it is possible to measure a variation in the impedance reading (schematic in Figure 4A).^37^

In this regard, an adhesive patch with tetrapolar electrodes (Figure 4B) was placed on the skin over the catheter, and the impedance was monitored.^37^ If the slope rate of the impedance over time showed a huge diversion, the power injector connected to the catheter was stopped. In the same study, two contrast agents, an ionic one (diatnizoate meglumine, Hypaque 60; Nycomed, Oslo, Norway) and a nonionic one (iohexol, Omnipaque 350; Nycomed) were tested on dogs. After 5 mL of extravasation of the contrast agents, the rate of the slope change per minute was 163% and 156%, for the ionic and the nonionic contrast agents, respectively. Notably, the impedance reading of the injection site is decreased if the fluid is ionic, while the impedance rises in the case of nonionic fluid (Figure 4C–D). After optimizing the shape of the patch by experiments on pigs, the same trials were performed on human patients where no noticeable extravasations were detected.^37^

Other experiments were carried out with human subjects to collect more information. Ionic and nonionic solutions were injected with different infusion rates of 0.25, 2.5, and 5 mL/s to human patients.^38^ A standard tetrapolar impedance measurement patch was placed on the patients’ skin, and the patch was connected to the power injector. The injector was programmed to stop the injection in case of a huge change in the flow rate. First, the baseline measurement was completed; then the injections of two contrast analogs, ionic (0.9% saline solution) and nonionic (5% dextrose),^37^ were carried out. After each injection, the extravasation size was inspected to check the occurrence of the extravasation event.^38^ The results exhibited that, for the flow rates of 2.5 and 5 mL/s, the sensitivity of the patch was 100%. However, at the flow rate of 0.25 mL/s, the device failed to give a signal to stop the injector in 11 out of 20 extravasation events. Infusion of the ionic fluid caused the impedance value to reduce significantly at any rate. On the other side, the impedance increase caused by the nonionic fluid was insignificant.^38^ At flow rates higher than 0.25 mL/s, the patch stopped the power injector at the average volume of 11.6 and 13.5 mL for the ionic and nonionic fluids, respectively. After remapping the injector’s algorithm to match the stored data for the case of the flow rate of 0.25 mL/s, an additional set of 10 patients was tested, and it succeeded in stopping the injector in 18 cases out of 20, at the average infused fluid volumes of 3.1 and 4.3 mL for ionic and nonionic fluids, respectively. In this study, it was noted that the baseline impedance for men and women are different and also that the modification of the algorithm was required especially for the low flow rate.^38^ The impedance-based sensor is now commercially available, for example, the Empower Injector System Extravasation Detection Patch (Bracco, Monroe Township, NJ, USA).

#### Temperature Sensor

Extravasation detection through temperature measurements is based on the fact that the extravasated liquid affects the temperature around the IV site. Since the measurement target is the surface of the skin, this method is noninvasive and requires external equipment such as a thermal camera.

**Figure 5:**
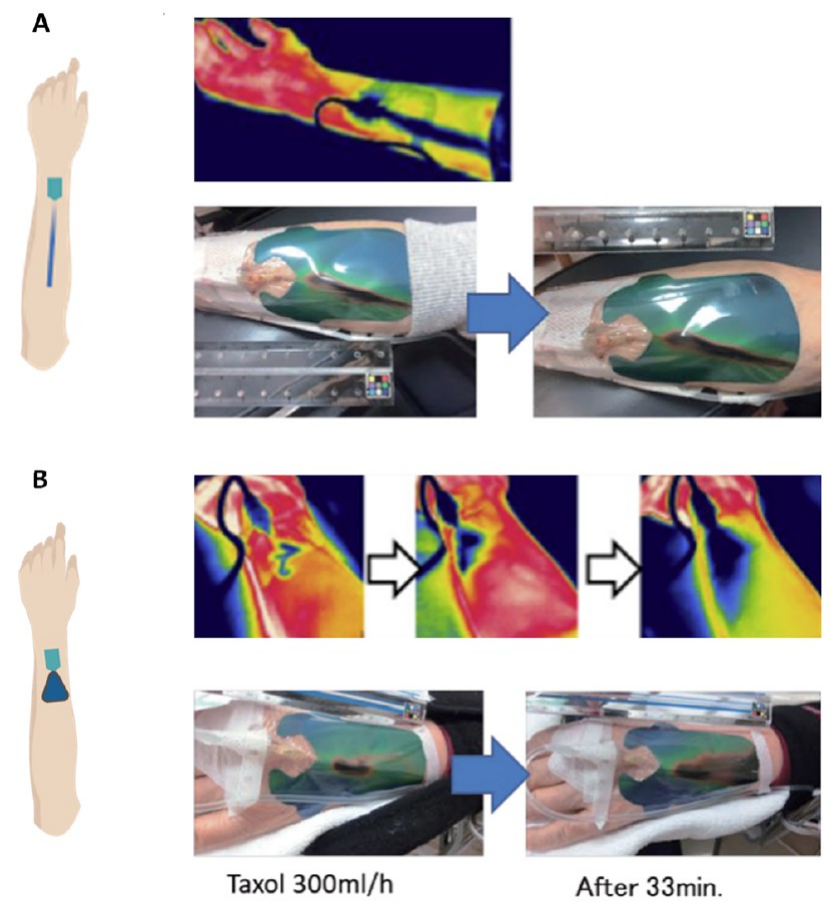
(A) The schematic, the pattern under the infrared thermography, and the patterns of the thermos-sensitive liquid crystal film, where no extravasation was observed. (B) The schematic and thermographic patterns under the infrared camera and of the thermosensitive liquid crystal film, where the extravasation event occurred. Infrared patterns reprinted with permission from ref (39). Copyright 2017 Elsevier. Patterns on the thermos-sensitive liquid crystal films adapted from ref (41) under open access license.

For a broader scope of the detection, infrared thermography was employed to measure the distribution of the skin temperature in the clinical setting for a real-time assessment.^39,40^ In a preliminary study, the thermography method was found to have a highly accurate detection sensitivity of about 85%.^40^ In this experiment, the thermographic pattern of the patient’s forearm during the injection was analyzed (Figure 5, the thermographic images under the infrared camera). To identify the extravasation condition, ultrasonography was performed before the catheter removal. From the investigation of the thermographic pattern, one pattern was correlated to the extravasation. This method was applied to the clinical test using a thermosensitive liquid crystal film during injection to visualize the pattern, which was aimed to simplify its use by the nurse (Figure 5, the patterns of the liquid crystal films). The thermosensitive liquid crystal film is inexpensive and requires no space and skills for the temperature detection. Furthermore, the film is not constrained by the patients’ movement. The pattern on the liquid crystal film was correlated to the infrared thermal camera, and it was proven that the pattern analysis on the liquid crystal film was valid for extravasation detection.^41^

#### Radiofrequency Sensor

**Figure 6:**
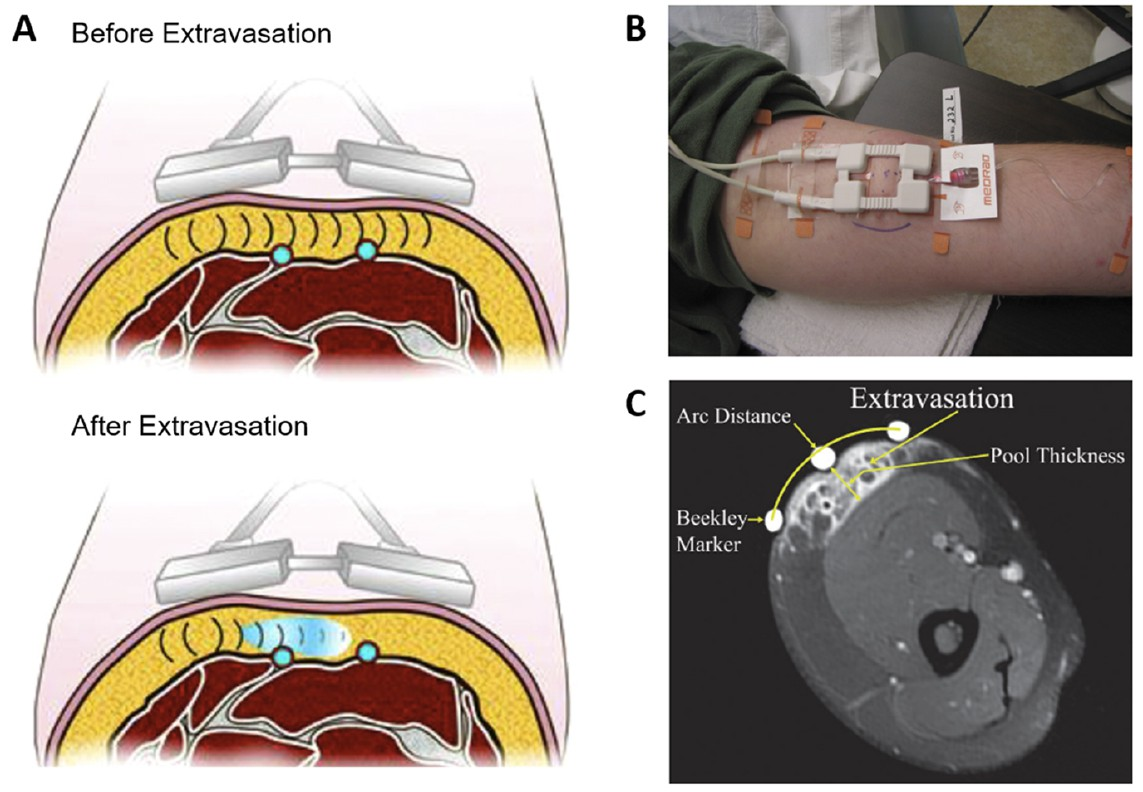
(A) Medrad XDS extravasation Detection System Operation of radiofrequency waves relative to veins. Top image (before extravasation) demonstrates the radiofrequency waves calibrating the tissue density before contrast administration; bottom image (extravasation) demonstrates the contrast extravasation being detected by changes in radiofrequency pulses from changes in tissue density. Reprinted with permission from ref (59). Copyright 2011 Elsevier. (B) A photograph of the radio frequency based extravasation detecting device and its placement. (C) Magnetic resonance scan (axis slice) of an induced extravasation. Reprinted with permission from ref (43). Copyright 2009 Wolters Kluwer Health.

Radiofrequency (RF) signals are employed to detect the change of the electrical permittivity of the soft tissue within the sensing range. A low-power radiowave is transmitted from the emitter through the tissue and received at the receiver.^42,43^ When extravasation occurs, the permittivity starts to change due to the huge difference of the relative permittivity between the tissue (typically 5) and the contrast agents (30–50).^57^ Due to this change, the propagation of the radiowave is affected about 10 fold (Figure 6A). The RF permittivity can differentiate the existence of many fluids (i.e., saline, contrast agent, and blood) and the system has a high sensitivity. Compared to some other techniques, the RF sensors do not require galvanic/conductive contact to the site. Furthermore, the RF method directly detects the signal amplitude where some other techniques monitor the time rate of the change. Also, the RF method is not affected by the depth^57^ or the temperature^58^ of the extravasation site.

In a study, Carr and co-workers^42^ developed a system that utilizes microwave monitoring. The detector was put on the patient’s arm during infusion to monitor the signal (Figure 6B) and following extravasation, the magnetic resonance scan of the arm of the axial slice was taken to observe the existence of the infusate (Figure 6C). The detection sensitivity of this method was assessed against the monitoring of the pressure as an alternative approach to detect the extravasated liquid, and the radiometry reading resulted to be more sensitive than the pressure monitoring. The results demonstrated that 7 and 3 mL of extravasation were detected in the dogs and the human subjects, respectively.^42^

Also, the effect of the patient’s posture to the extravasation detection by RF sensor was tested. A total of 65 induced extravasation was performed with an RF sensor. A prototype unit of the sensor (Medrad Patient Sensor Assembly, Model PSA 700) was placed on the patient’s arm with different postures and the catheter was intentionally placed outside of the vein. Twenty milliliters of saline solution at the flow rates of 0.1 to 4 mL/s was infused. The threshold for the false alarm was set to 0.1% and 65 out of 65 extravasations were detected. No false-positives were observed, regardless of the movement of the patients. The estimated sensitivity for the system was 99.8%, and the estimated specificity was 99.97%.

#### Optical Sensor

**Figure 7:**
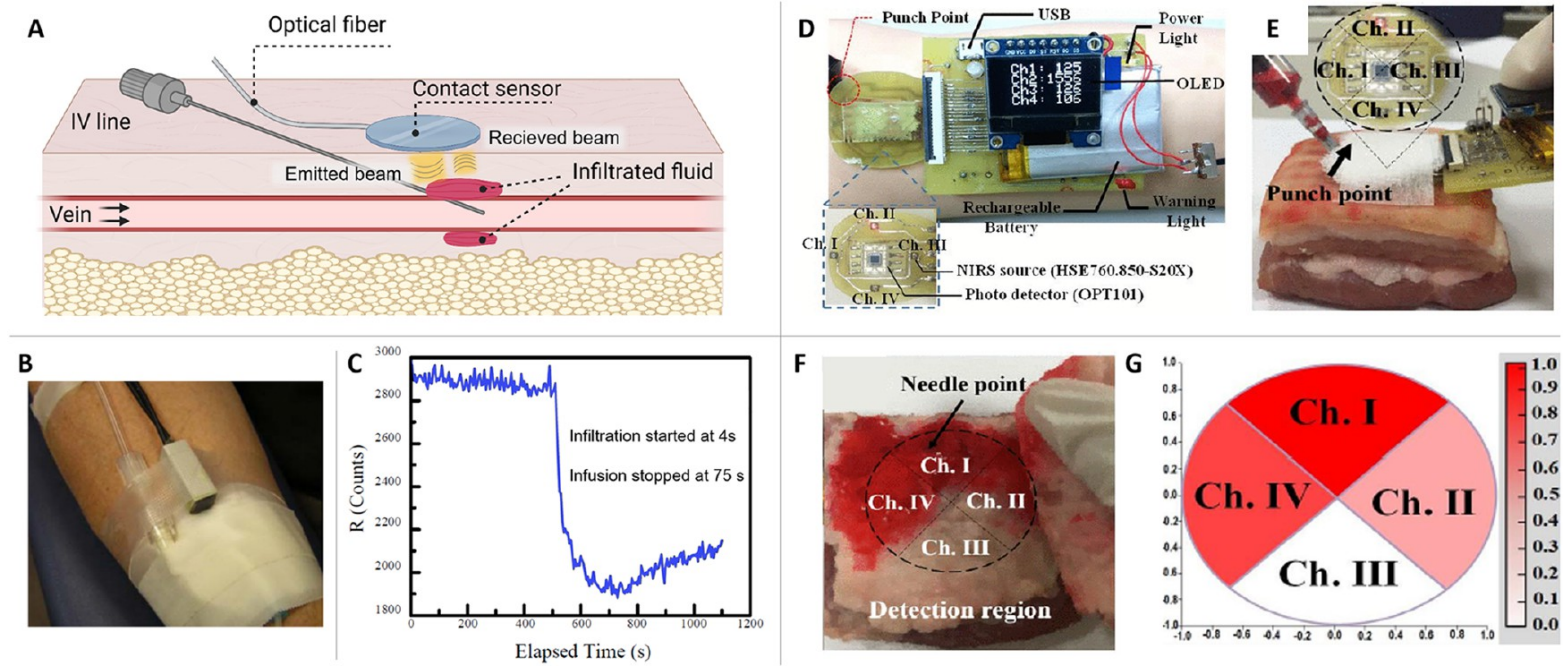
(A) A schematic of the extravasation detection mechanism based on the optical emitter and receiver. (B) An actual fiber sensor with an IV catheter on a patient’s arm. Reprinted with permission from ref (45). Copyright 2008 IEEE. (C) A typical signal radiation value against elapsed time with the extravasation induced. Reprinted with permission from ref (44). Copyright 2006 SPIE. (D) An example of optical sensors as extravasation detection device, comprising four near-infrared light transmitters and a photo detector used to construct an array sensing system. (E) The device from (D) tested on an actual pork tissue, where the free injection was induced. (F) The pork subcutaneous tissues after induced extravasation compared with (G) the two-dimensional distribution map reconstructed real time by measurements from the device. Reprinted with permission from ref (47). Copyright 2017 IEEE.

Optical sensors consist of a beam emitter and a beam receiver, which are placed on the skin. The beam is emitted and goes through the catheterization site, being reflected, scattered, and diffused, then is detected by the receiver (Figure 7A).^44^ When an extravasation incident occurs, the fluid in the soft tissue changes the optical density of the beam and the reading of the beam receiver. The value at the receiver is compared to the baseline value that is performed before the extravasation, and then, a certain threshold is set to define an extravasation.^44^

The sensor was tested in the animal experiments with infusion of saline with an infusion pump.^44^ The extravasation occurrence was confirmed by visual inspection, spectral characteristics of a fluorescein marker, and/or the absence of blood return in IV line. Extravasation simulations were performed on pigs for 37 times. Fluid was injected at increase of 0.1–0.25 mL at a time with 0.5–2.0 min interval, or with an infusion pump at 3–10 mL/h. A weal formed by extravasation generated increase to signal radiation, due to that the skin layer moves closer to the contact sensor or decrease because the skin volume was increased. In all cases, the extravasation was detected before the weal was formed. In the simulated extravasation experiment, the smallest fluid volume to detect was about 0.1 mL for manual injection using syringe and 0.02 mL when syringe pump or infusion pump was used. Only one false-positive and one false negative were detected. For threshold of the reading value difference of 5% and 10%, 97% prediction were achieved for positive and negative results. 65 induced extravasation experiments on pigs were performed. After infusing the fluid for 5 min to 1 h at the flow rate of 3–10 mL/h, extravasation was intentionally started. After the sensor detected the extravasation, the pump was turned off, and the reading was recorded for more minutes to detect the fluid diffusion. In the typical cases, the reading value decreased for 10% for a period of 20 s. After extravasation was stopped, the reading value gradually increased (Figure 7C). The minimum detectable volume of the fluid is estimated around 0.1 mL. Finally, the trials were done on 51 human volunteers and the sensor’s detection ability was compared to fluorescein marker and visual/tactile detection by nurses. The sensor detected the extravasation much earlier than the nurses. For the visual inspection, sometimes the extravasation was confirmed by the typical color of the fluorescein (yellowish orange) by the nurse, therefore in case the fluid is transparent only the sensor could detect the occurrence. The most likely sensitivity and specificity were estimated to be 0.93 and 0.95.^44^ A device utilizing this sensor (Figure 7B) was developed and connected to the wireless network. The sensor readings were stored on PDA at hand of the clinician Figure 7.^45^

To correlate the optical sensor readings and actual CT scan image, extravasation simulation using contrast agent was done on 7 swine models.^46^ The contrast agent was injected at the rate of 1.0 mL/s and the light intensity from the sensor was read and compared to the baseline. When the sensor reading exceeded the limit, the injection was stopped. The detection duration and volume of the extravasated fluid were determined. After the extravasation was detected, CT scan image of the injected area with the sensor attached was taken and the depth was calculated. Minimum detectable extravasated volume was 0.9 mL in 2.0 s. The detection time varied among the samples and the longer the detection duration, the larger the extravasated volume was and the deeper extravasation volume. No false positives or false-negatives were observed.^46^

Du et al.^47^ developed the array sensors of near-infrared to detect the fluid extravasation by the comparing different locations (Figure 7D). The experiments were conducted on pork tissue with epidermis, dermis, subcutaneous tissue, fat and muscle layer. For the injection fluid, venous pig blood with high concentration of deoxygenated blood was used. First, the sensors were tested to see the extravasation sensitivity. The sensor array was attached to the pork tissue (Figure 7E), then initial values were read. 0.5 mL of the pig blood was dropped on each sensor and the values were read. All the four sensors responded to the dripped blood and no cross-sensing was observed. Second, a free injection testing was conducted to observe the blood diffusion (Figure 7F). After injecting and reading were done, the pork was cut to visually compare the blood diffusion and the sensors readings. The blood diffusion in the pork tissue matched to the sensor readings (Figure 7G). The detectable depth was 2 cm and minimum volume was 0.3 mL.^47^

#### Ultrasound Sensors

Ultrasonographic detection of extravasation refers to the visualization of the position of the vein and the syringe/catheter tip in the patient’s body before and during the injection is conducted. The injection site is observed with an ultrasound scope and the gray scale ultrasonographic image is shown on a monitor, so the clinician can dynamically see the position. A few cases are reported for neonates and pediatric patients.^60,61^ Here, more established methods for the needle and catheter positioning are introduced.

In a study, the color-flow injection analysis was conducted to ascertain the catheter positioning and it was compared to the standard position confirmation tests.^48^ The color-flow injection test was performed in the following procedure: 1 mL of preservative free normal saline was injected within 2 s into the intravenous catheter and the turbulence in proximal draining veins of the corresponding limb were observed during the injection by the changes of the hue of the color pattern. The primary end point was the change in flow in the proximal draining veins with rapid flush of normal saline. The catheters were visually inspected for extravasation after the procedure. The color-flow injection test showed a sensitivity of 100% and specificity of 100% to confirm the correct catheter positioning, while where standard confirmation tests were used, the highest sensitivity was 88% (due to the presence of smooth injection).^48^

**Figure 8:**
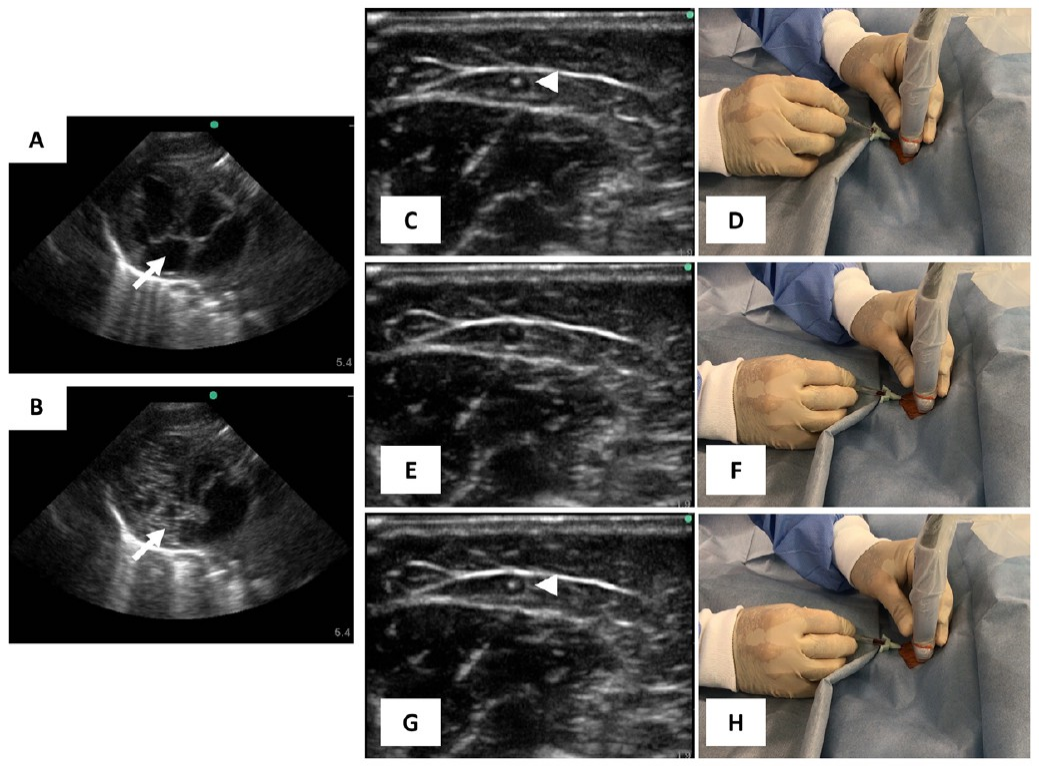
(A, B) Extravasation detection based on microbubble detection test through ultrasounds: (A) parasternal four-chamber view before saline injection. The arrow points the right atrium and (B) parasternal four-chamber view with the microbubble detected in the right atrium, shown by the arrow, after saline injection. Reprinted with permission from ref (49). Copyright 2019 Wolters Kluwer Health. (C–H) Ultrasound-guided insertion of catheters to avoid extravasation. Confirmation of the needle position by ultrasonography (C, E, G) and corresponding hand positions (D, F, H). (C, D) The needle tip is confirmed within the vein (arrow). (E, F) The needle tip disappears from the view by adjusting the insertion angle. (G, H) The needle was advanced and appeared on the screen again. Reprinted with permission from ref (62). Copyright 2020 Elsevier.

Extravasation detection with microbubble detection test was performed with 137 pediatric patients and the sensitivity and specificity were compared to the smooth saline injection test.^49^ The microbubble detection test procedure is as following: 10 mL normal saline was injected within 2 s through an infusion line connected to a catheter using a prefilled syringe. Simultaneously, the parasternal or epigastric four-chamber was shown ultrasonographically (Figure 8A, B). The microbubbles present in the saline injected were visualized in the right atrium by ultrasonography. If microbubble turbulence was visible within a few seconds of the injection, the test was considered as positive, otherwise negative. The sensitivities of the microbubble detection and the smooth saline injection test were 100% and 89%, respectively. The specificity of the microbubble detection and the smooth saline injection test were 100% and 64%, respectively. The microbubble test showed much higher values for both.^49^

To confirm the placement of the needle and the cannula, Takeshita et al.^62^ applied dynamic needle tip positioning (DNTP) method. In this method, both the needle tip and the ultrasound probe were advanced alternately using the short axis out-of-plane approach.^49^ With the ultrasound transducer, the needle tip inserted in the patient’s body was seen as a bright dot on the ultrasound screen (Figure 8C, D). Then, the transducer was moved to the proximal direction until the needle tip disappeared from the screen (Figure 8E, F); then, the needle tip was advanced until it appeared on the screen again (Figure 8G, H). The sequence was repeated until the needle tip punctured the anterior wall of the target vein, and the blood return in the catheter hub was confirmed. The overall success rates of the catheter placement was 97.5% (*n* = 39).^62^

#### Fluid Pressure and Resistance

The extravasation was analyzed in real time from the perspective of the fluid pressure. Specifically, when the fluid does not flow in the vein, the pressure of the fluid, or the fluid resistance increases. Based on this, the prediction of the extravasation was proposed theoretically;^63^ the model for the fluid administration to intravenous systems and human subjects was studied, and several pressure-flow models were proposed.^63,64^ In addition, the pressure-flow relationship (PFR) was analyzed before and during the infusion. By observing the PFR in before and after the catheter placement, it made clear that the flow resistance of the vein is lower than the flow resistance of the tissue.^65^

**Figure 9:**
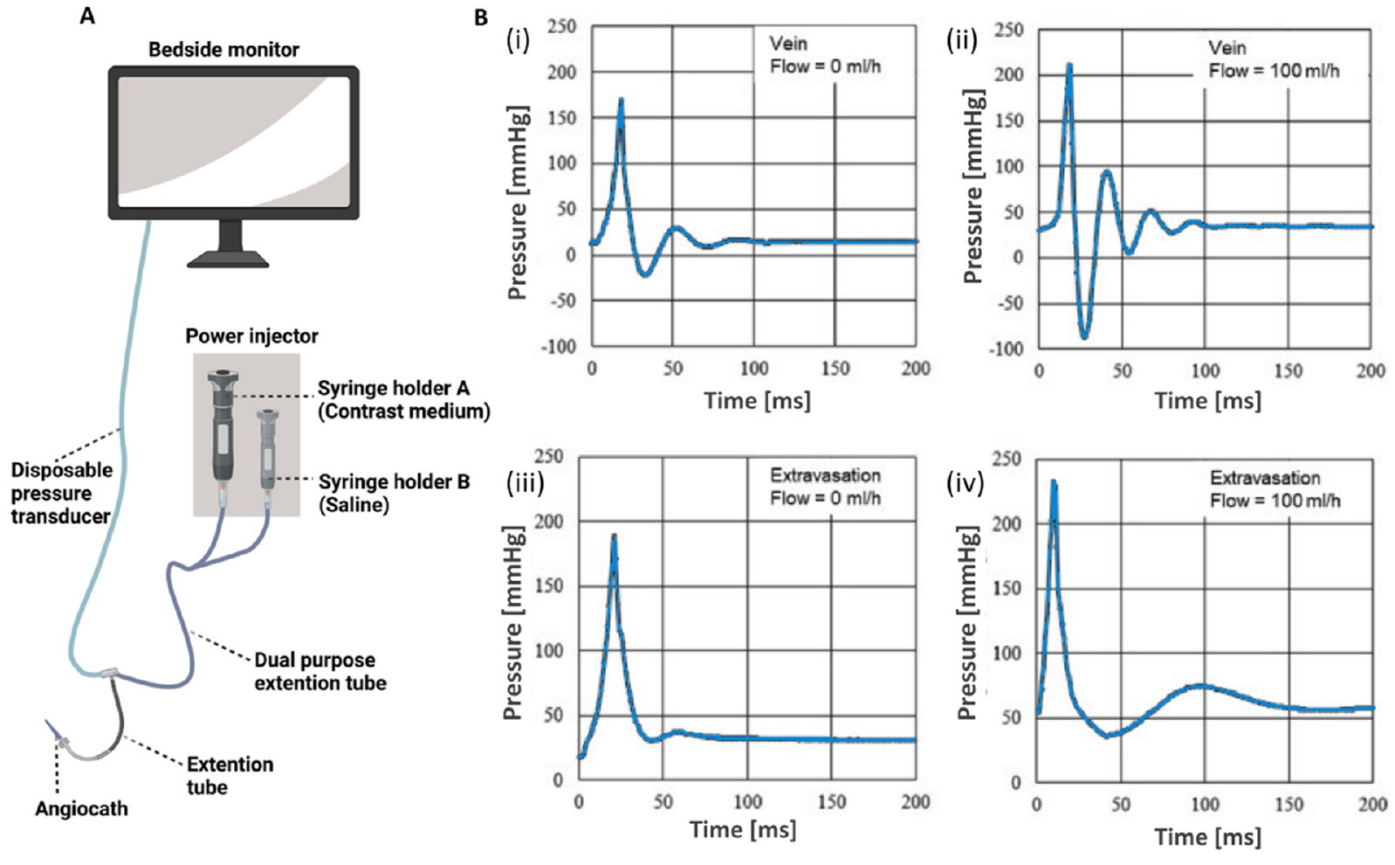
(A) Experimental setup for measuring the pressure generated in the extension tube by using a disposable pressure transducer during the power injection of saline and contrast medium. Reprinted with permission from ref (50). Copyright 2013 Springer Nature. (B) The impulse-oscillometric response of a catheter-sensor-system used as a way to detect the onset of extravasations by differentiating the venous and extravasational catheter placement. The impulse-oscillometric pressure response at the peripheral vein catheter for venous placement without (i) and with (ii) infusion liquid flow result in different trends compared to the pressure oscillations at the peripheral vein catheter for extravasational placement without (iii) and with (iv) infusion liquid flow. Reprinted with permission from ref (67). Copyright 2021 Elsevier.

Abe et al.^50^ proposed a new injection program named “saline test injection mode” for the use of a power injector able to adjust the injection mode to a specific rate and volume and to predict the reaction to contrast agent administration. Contrast medium extravasation occurred, and body movement was invoked by the stimulation from the injection site; therefore before the contrast agent injection, the equivalent amount of saline was injected. During the injection, the change of the pressure was monitored to detect any possible abnormality (Figure 9A). The relationship between the injection pressure of saline and contrast agent was investigated in the phantom model with various injection rate. From the investigation of the test in 473 patients, side effects were detected in 21 (4.4%) out of 473, extravasation in 5 (1.1%), high pressure in 7 (1.5%), and stimulation in 9 (1.9%). After the failure in the saline injection test mode, the connection was rerouted and the contrast agent was administered. However, the patients showed a stimulation response from the contrast agent injection and the examination failed.^50^

As an alternative approach, the impulse-oscillometric response of a catheter-sensor-system (CSS)^66^ was applied to detect the onset of extravasations. The feasibility of the impulse-oscillometric response method to detect extravasation was tested.^67^ For the ex vivo experiments, the shank (crus) of a pig was used and the *vena tibialis posterior* and the musculus triceps surae, were punctured, for the venous catheter placement and the extravasational placement, respectively. The vein and the CSS were prefilled with distilled water, and the 100 mL/h rate was generated by an infusion pump. The pinch valve generated a flow impulse, while compressing the infusion line. The infusion line was not completely occluded to obtain ongoing fluid delivery and to prevent the formation of a bolus in the infusion line. Pressure trends were recorded for two seconds between closing and opening of the valve. The impulse generated by the pinch valve induced a strong rise in pressure at the peripheral vein catheter. After reaching a peak, the pressure trend performed a damped oscillation (Figure 9B). Without (Figure 9B(i), Figure 9B(iii)) and with (Figure 9B(ii), Figure 9B(iv)) flow, significant differences for the parameters, such as the frequency, averaged maximum amplitude, damping, decay constant, were observed. The system could be applied to the extravasation detection before and during the infusion.^67^

#### Pressure Sensors—Skin Deformation

**Figure 10:**
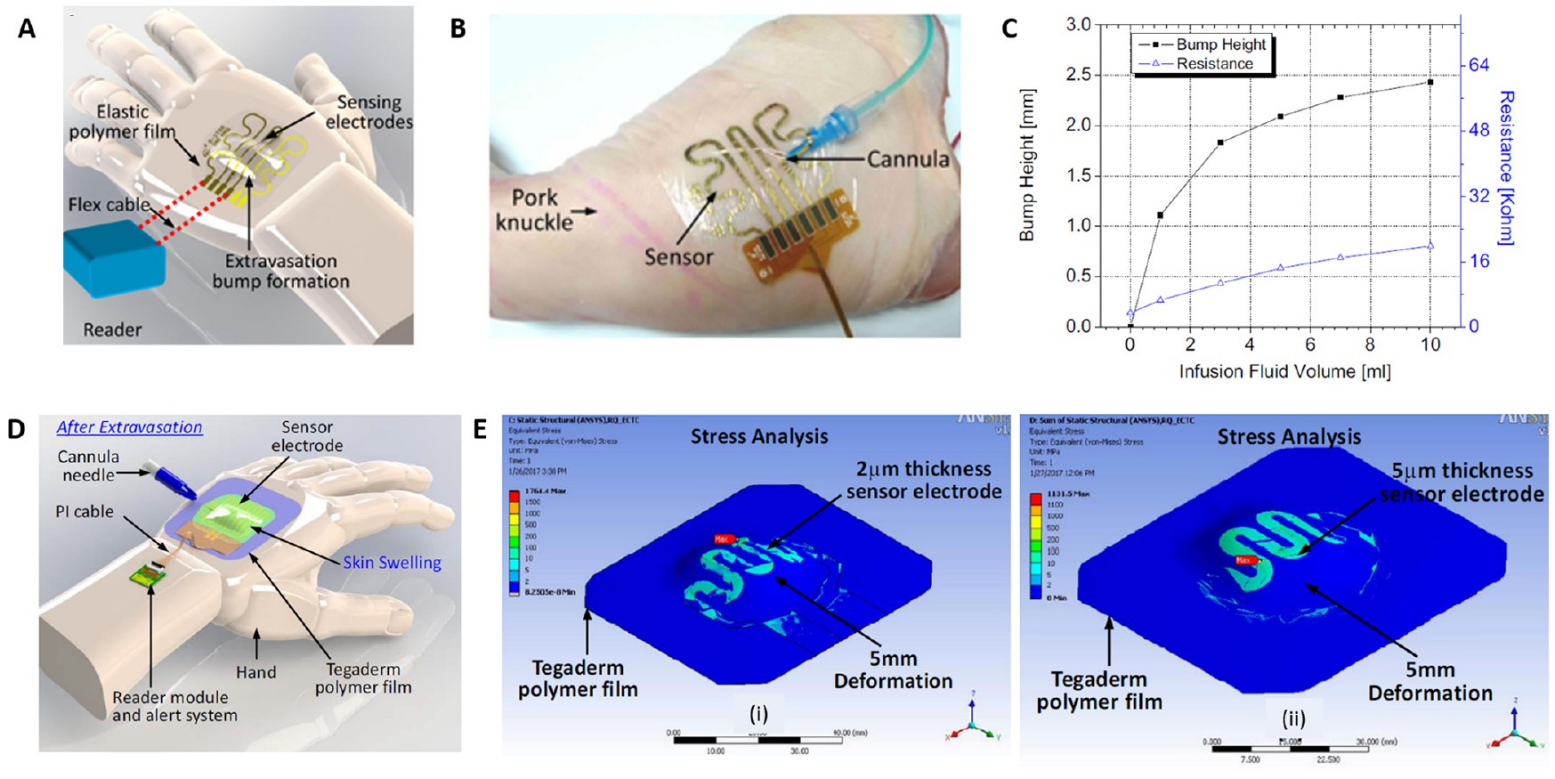
(A) Schematic of the proposed sensor for extravasation injury detection. (B) Ex vivo analysis of the attached sensor on the pork knuckle. (C) Bench-top test result of the fabricated sensor. Reprinted with permission from ref (16). Copyright 2016 IEEE. (D) Schematic of sensor patch system for skin swelling detection. (E) Strain simulation result for 2 μm thick sensor electrode. Reprinted with permission from ref (51). Copyright 2017 IEEE.

The skin pressure/strain sensors are based on the bumps or swellings that are created by the extravasated fluid in the soft tissue. The sensors consist of several electrodes integrated into a soft material and attached directly to the skin, on the injection site. When a bump is formed by extravasation, the distance of each electrode pair is increased and the impedance value changes. In this regard, strain sensors were fabricated to detect skin deformation. The sensors were patterned on adhesive wound dressing films by thermal evaporation of Ti/Au through a shadow mask (Figure 10A).^16^ For the benchtop experiment, the sensor was attached to the fixture and deformed by compressed air, mimicking the bump formed by extravasation. The resistance value from the sensor and the height of the bump formed by the air were recorded simultaneously, and from the result the algorithm to detect the extravasation was determined. The ex vivo test was conducted with a pork knuckle (Figure 10B). The flow rate was fixed at 50 mL/h and contrast solution was injected. It was concluded that minimum detectable extravasated fluid was less than 5 mL (Figure 10C).^16^

With a similar configuration, a thin film metal sensor was designed and structural simulation analysis was conducted. ANSYS finite element analysis was used with the three-dimensional models of the patch, thickness of 2 and 5 μm, with the boundary conditions (Figure 10D–E).^51^ A deformation by a spherical object, of diameter 20 mm and 5 mm *z*-directional deflection was applied. The deformation along the electrode and the film was analyzed. The difference of the thickness did not have effect on the strains. The designed device was fabricated on a polymer film by sputtering Ti/Cu/Au of the thicknesses of 20 nm/2 μm/20 nm via a shadow mask on a polymer film. Double-sided conductive films were attached to the electrodes as wires. The tensile test showed that the polymer film used as the substrate in the device had a break point at 1345%. Then the sensor underwent the ex vivo test with a pork knuckle. The sensor was attached to a pork knuckle and the catheter was inserted. Fluid of water and food dye was flown by a syringe pump at 50 mL/h, while the formation of the bump was observed by a 3D camera. The sensor reading and the bump height was measured at every 1 mL infusion. From the result, the minimum detectable extravasated volume was determined as at least 2 mL, which caused the formation of the bump with the height of 4 mm.^51^

**Figure 11:**
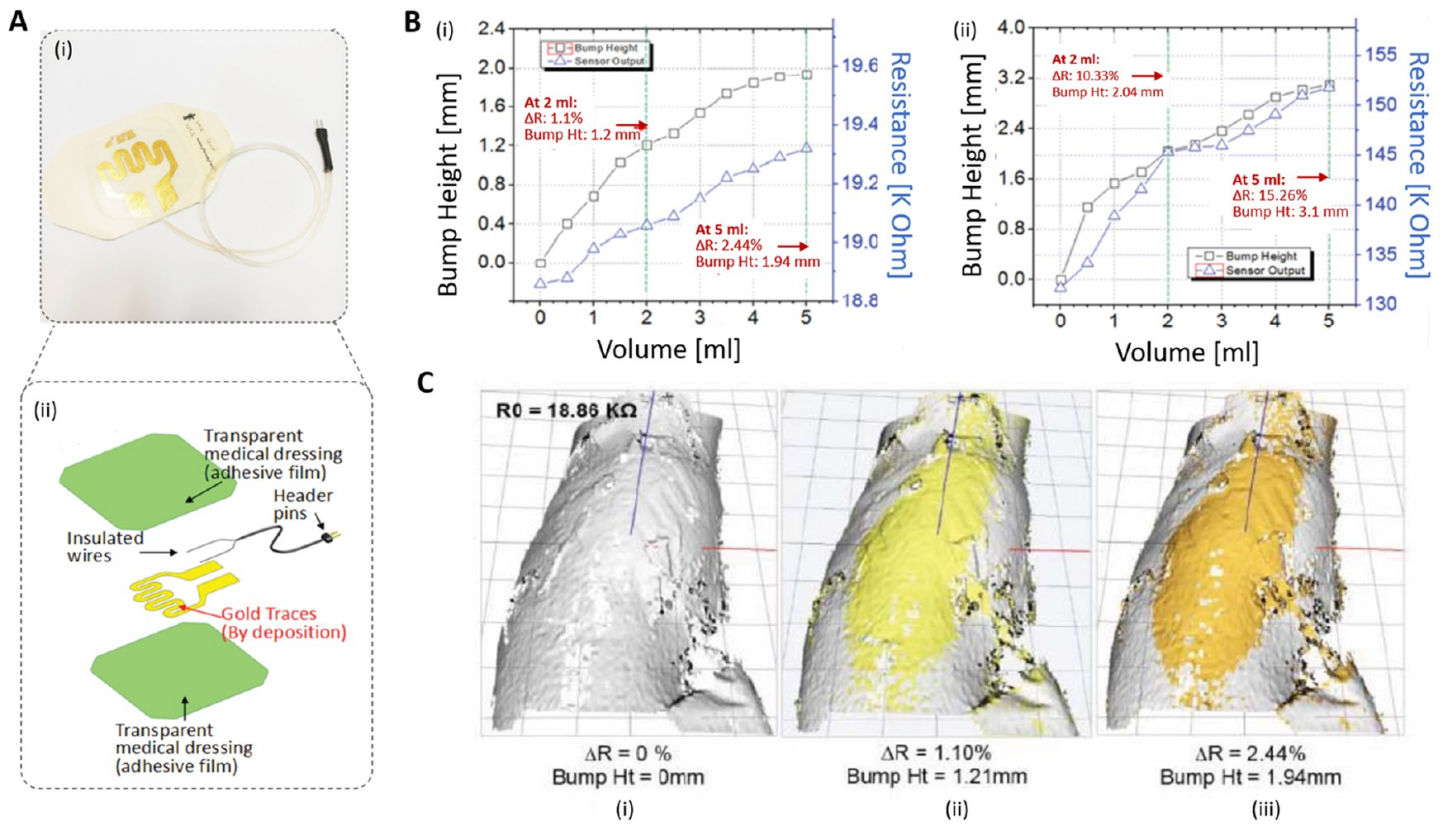
(A) (i) Developed sensor patch shows the gold traces by metal deposition and (ii) the exploded view of the sensor patch showing that the Au traces are embedded in the adhesive film. (B) Result of the ex-vivo test: bump height and resistance of polymer-based carbon traces against infused volume (i) using the existing 3 mm design and (ii) using narrow traces. (C) Representation of the 3D scans from ex-vivo test conducted using the polymer-based sensor patch that have the exact same design as the gold patch. Adapted with permission from ref (52). Copyright 2018 IEEE.

The same group realized a strain sensing skin patch with screen-printed polymer-based carbon with a similar design as the previous one (Figures 10 and 11A). The polymer-based carbon black is advantageous compared to Au in terms of cost, manufacture, and sensitivity.^52^ To increase the sensitivity, two types of the sensor that were embedded between two adhesive films, and one type that was fabricated on a single adhesive film, with two different electrode designs were prepared. From the benchtop test, the electrode pattern printed with polymer-based carbon on single adhesive film showed higher sensitivity result than the original electrode design in the previous work.^51^ The samples underwent the ex vivo test with pork knuckle. The printed carbon sensors, however, showed less sensitivity than the Au electrodes. The sensitivity to the 5 mL infusion with 1.94 mm bump height was only 2.44% for the carbon-based sensor, where it was 40% for Au-based sensor (Figure 11B, C). This was considered due to the fast diffusion of the infused fluid because of the variation of the cut of the pork sample.^52^

The Au-based patch and the carbon-based patch were further tested for in vivo experiments with a piglet model. Up to 6 mL of saline solution were infused. The sensor patch detected less than 2 mL infusion for early extravasation detection with no false negatives. The bump height and the sensor reading were compared and the clear correlation to the two values were confirmed. The Au-based patch was also used for clinical trial to stimulate extravasation conditions. Nine healthy volunteers were treated with local anesthetic cream to the punctuation site, and the catheter was placed deliberately out of the vein. The sensor patch detected less than 2 mL infusion for the early detection. The resistance reading increased exponentially and with 2 mL of infusion resulted in higher than 40% resistance than the initial value. For 9 participants, no false negatives were detected. The formed bump heights were between 2.1 and 4.4 mm, which were not clear under visual inspection.^53^

Instead of the use of carbon or Au, a biocompatible conductive polymer was also used for the sensor. The electrode pattern was printed on a transparent film dressing using two inks; poly(3,4-ethylenedioxythiophene) polystyrenesulfonate (PEDOT:PSS) dispersion with polyethylene glycol (PEG) additive and Ag nanowire with diethylene glycol (DEG). The inks were patterned using an ink dispensing system. The patterned electrodes were used as the bottom electrodes and combined with the top electrode of an indium tin oxide coated polyethylene terephthalate (ITO/PET) conductive film. After assembling the device, the correlation between the pressure given to the sensor and the resistance reading were compared. As the pressure was applied, the two electrodes were compressed and the electrical contact resistance decreased.^54^

#### Gamma Scintillation Sensors

**Figure 12:**
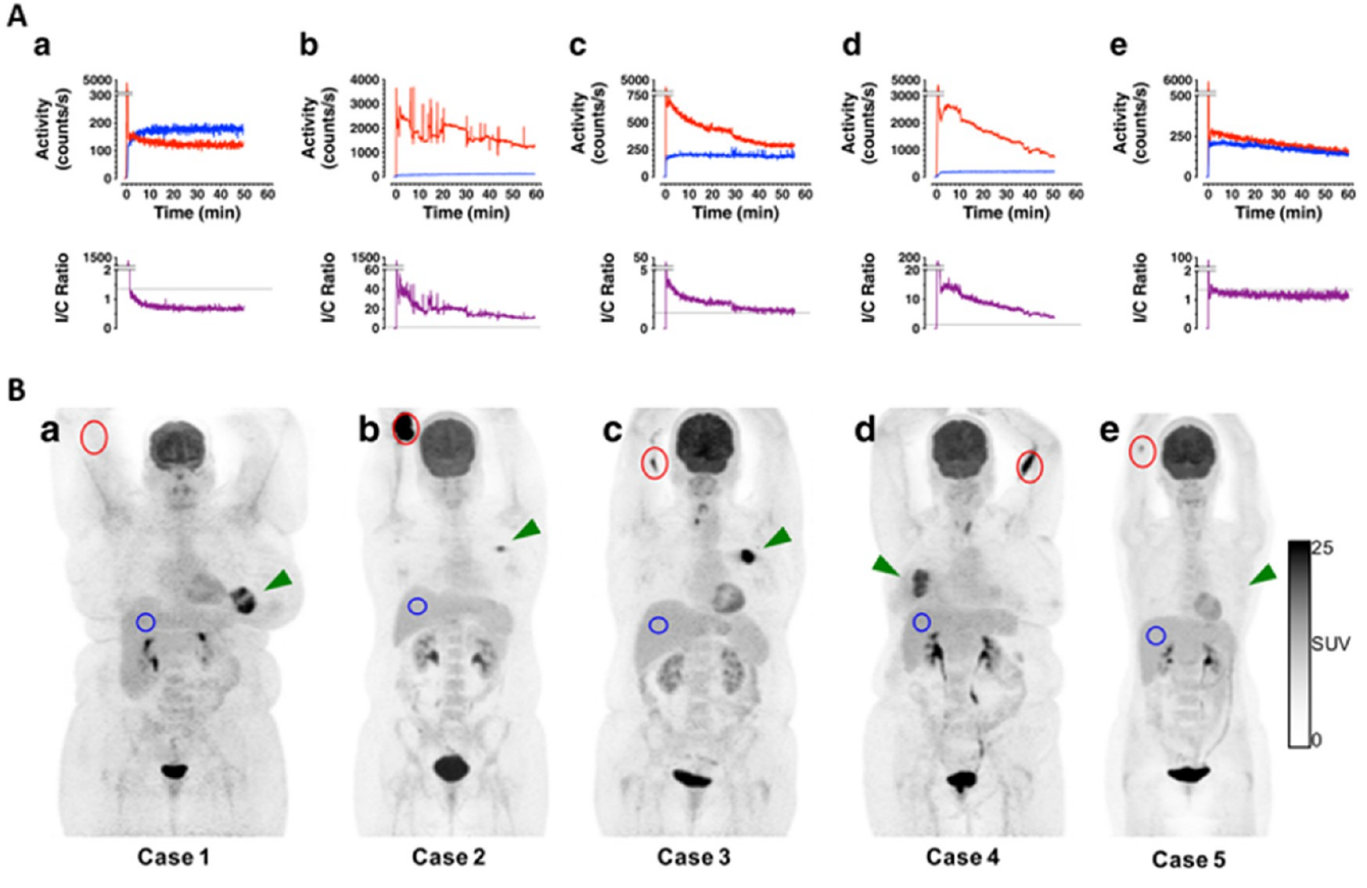
(A) Time–activity curves (TACs) from sensors placed over the injection arm (red) were normalized to those obtained on the control arm (blue) to determine the I/C ratio (purple) in a patient with no visible infiltration (a) and patients with severe (b), moderate that resolves to small (c), and severe that resolves to moderate (d) infiltration. (B) Maximum intensity projections from PET images, corresponding to the sensor time–activity curves (TACs) in (A). One patient (A-e) had a low activity spot of FDG, visible on PET (B-e), but the time–activity curve data were inconclusive due to sensor distance from this spot. Note the differential ordinate scaling. Reprinted with permission from ref (55). Copyright 2016 Springer Nature.

When the injected medium is radioactive, the flow of the medium can be detected via gamma scintillation sensors. Having this in mind, 3 topical scintillation sensors were placed on different locations, i.e., over the tumor, over the injection arm, and over the control arm. The sensor values were recorded during ^18^F-fluorodeoxyglucose (FDG) was injected for PET (positron emission tomography)/CT examination and the extravasation was confirmed via PET images. From the readings of the sensors on the arms, the injection/control (I/C) ratio was calculated (Figure 12A). When the patient had a severe extravasation, the injection/control value showed values much larger than 1, though the value and the shape differed by the patient. For comparison, maximum intensity projections were obtained from PET and the concordance with the signal readings was confirmed (Figure 12B).^55^

### Multimodal Sensing

Multimodal sensing, or sensor fusion, refers to the use of several different sensors to detect the phenomenon from various aspects.^19,43,68−71^ As several sensor systems have been introduced earlier, the multimodal sensors could complement each other’s disadvantages and generate synergetic effect for the sensing.

Jambulingam et al. developed multimodal sensing system using skin-stretch and bioimpedance measurements. The system was tested on pork belly for benchtop ex-vivo. The bioimpedance at the extravasation site was changed from 815 Ω to 795 Ω by 3 mL infusion, which was within the measurable range by the system. Also, the strain value increased by 2.25% after 3 mL infusion. The timings of the two changes were associated in the in vivo experiment.^68^

In another study, Bicen et al. applied the bioimpedance measurement from the previous work^68^ and skin stretch sensor. Bioimpedance was measured using Ag/AgCl wet electrodes in a tetrapolar configuration, while a strain sensor was coated with silicone and applied for the detection of skin swelling. Acquired signals were processed and went through a decision table, where the extravasation was determined. An in vivo experiment with a pig model was conducted. IV infusion was done at 0.05 mL/s, and normal infusion and intentional extravasation of 2 mL, 5 mL, 10 mL, and an additional 10 mL of 0.9% saline solution were conducted. Under the condition of false alarm probability of 0.05, the decision variable statistics were studied. The detection probability by the bioimpedance for 10 mL infiltration was greater than 0.9 and that for skin strain sensing of 2 mL and the additional 10 mL. The combination of the result of the bioimpedance and skin stretching should consider the infusion volume and subject-specific characteristics, such as skin elasticity and skin impedance.^19^

**Figure 13:**
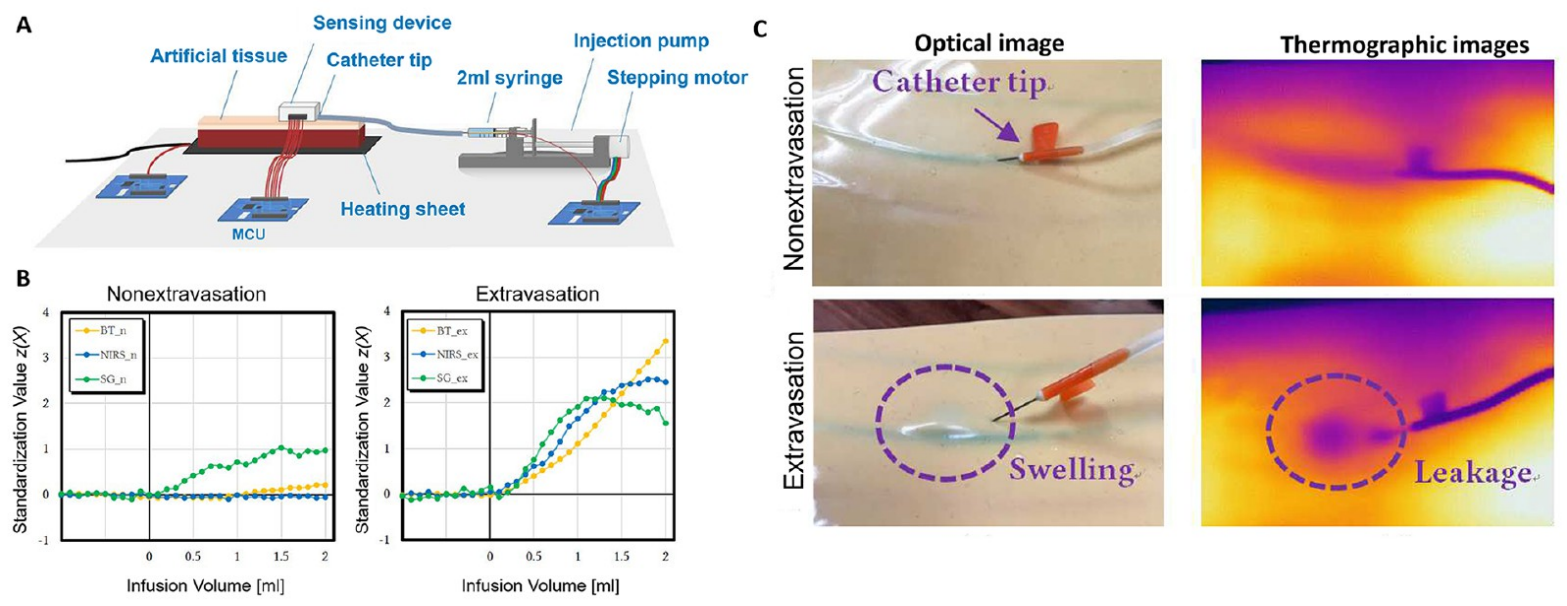
(A) A schematic of the device. (B) Sensor readings by infusing different volume of the induced infusions. (C) Thermographic images from artificial tissue in extravasation and nonextravasation infusion; the darker color indicates lower temperature. Reprinted with permission from ref (72). Copyright 2019 the authors.

A sensor-fusion detection platform was fabricated to distinguish the early signs of extravasation (Figure 13). In detail, a hybrid system of the body temperature, near-infrared spectroscopy, and strain gauge was developed and tested on an artificial skin model (Figure 13A). By the occurrence of the extravasation, sensor readings from all 3 sensors increased significantly (Figure 13B).^72^

The sensor was further expanded to integrate an optical sensor to monitor the fluid volume in the tissue and integrated onto a single board. On the testing platform, two syringes, one for a normal infusion inside the artificial vein at the flow rate of 10–60 mL/h and the other placed at the outside of the vein for the extravasation at the flow rate of 1–15 mL/h, were connected to the artificial skin model, and both are independently and remotely controlled without changing the location of the catheter tip (Figure 13C). To induce some randomness to the test, the artificial skin was placed on the arm of 30 human subjects and the extravasation was simulated. The collected data by the sensors were processed with deep learning techniques, including convolutional neural network (CNN) and long short-term memory (LSTM). Among the combination of the input of the sensors and the three analytical model proposed, analyzing all the sensors on board using the one mode had a high detection rate of 83.7% and the false alarm rate of 6.2%.^17^

## Concluding Remarks, Challenges, and Perspectives

Peripheral intravenous cannulation is widely applied in hospitals and medical clinics. However, the success rate of this procedure is highly dependent on the experience of the practitioner and the patients’ features, often leading to an adverse event known as extravasation.^36,69^ In this article, an overview of the extravasation mechanism and of the potential issues the patients might experience as a result of such an incident were represented. Many studies in the literature report that, to date, visual inspection of the cannulation site by physicians and nurses is the most common method used to detect extravasation.^44,73^ However, this strategy strongly depends on the experience of the physician himself and also on the time he could devote to constantly observe the patients—that is an issue especially in the case of infusions on a prolonged period of time. In any case, the extravasation event could be even detected long after its onset, especially if it occurs deep in the tissues. Moreover, the extravasation detection devices on the market are bulky and expensive, mainly developed to monitor contrast injections in CT scan patients.^16,74^ Given this, it is evident how important it is to develop technological solutions that can prevent or, at least, detect the onset of the extravasation during IV procedure.

We have highlighted here the latest and most advanced devices that were fabricated for extravasation detection, classified according to their operating principle. Impedance sensors consist in general of a patch to be placed on the skin integrating a couple of electrodes generating alternating current flows and another pair of electrodes reading the impedance value. These sensors are able to identify extravasation events based on the increase/decrease—depending on the type of fluid injected—of the measured impedance value and can reach 100% sensitivity in a specific flow range (2.5–5 mL/s). Temperature sensors detect a variation of temperature around the IV site due to the extravasated liquid. This type of noninvasive technology appears to be promising especially when used with a thermosensitive film to be placed around the catheterization site, instead of using a thermal imaging camera which is bulky and could restrict patients’ movements. Radiofrequency sensing technologies base their operating principle on changes in electrical permittivity when the extravasation event occurs. The extravasation event can also be detected by using an emitter beam that is reflected, scattered, and diffused at the level of the catheterization site, arriving at the receiver. We talk in this sense about optical sensors. The reading of the receiver changes the presence of extravasated fluid due to some changes in the optical density. Also, fluid pressure could be a good indicator for extravasation detection as the liquid does not flow smoothly in the event of a blockage. Anyhow, in this case, no significant advantages were observed over the other methods presented. Two other methods for extravasation detection consist in the monitoring of the bumps and swellings of the patient’s skin around the catheterization site by using strain sensors or scintillation sensors. However, the use of the latter is limited to radioactive media.

All the reviewed devices present steps forward to overcome some of the challenges related to the detection of extravasation, and they all are sources of inspiration for the development of a highly sensitive and comfortable (both for the patients and physicians) device. The use of wearable electronic devices is of course of great interest so as to replace the most used commercial bulky devices. Thin patch-based devices to be placed on the cannulation site allow the patient to remain in comfort and without limitations in its movements. Many strain-gauge biosensors are in the shape of wearable electrodes, and the examples reported here present the important feature of being transparent: this aspect is important in order to be able to continuously monitor and visualize the cannulation site. In parallel, these types of systems are perfectly suited to the possibility of exploiting rapid prototyping manufacturing techniques for their development.

In this review, we highlighted several times how difficult it is to prevent extravasation. If it is not possible to completely prevent this phenomenon, it could be, therefore, useful to be able to reduce the minimum amount of detectable volume by the sensor. In this regard, during the implementation of the device, it is important to minimize the detected volume, which represents the sensitivity of the device. By comparing the reviewed sensors, it turned out that strain-gauge biosensors, despite their conformability to the skin and their ability to be attached/detached, have too large minimum detection volume of leakage compared to others, showing low sensitivity.

As introduced earlier the big challenge is to detect early extravasation, just at its onset, in order to avoid complications in the tissues due to the leaked infused fluid. Optical sensors seem to be promising in addressing this issue since human experiments have shown that the sensor can detect extravasation earlier than nurses. Anyhow, their sensitivity is not as high as in other cases, i.e., impedance or radiofrequency sensing technologies emerged to have the highest sensitivity, and this could of course limit their application. Notably, contrary to impedance sensors, radiofrequency-based devices do not depend on the depth at which extravasation occurs. Furthermore, this is the only method among those reviewed here that allows for detecting the actual amplitude of the signal instead of the temporal frequency of the change on which other technologies are based. However, impedance sensors still present a high level of sensitivity. On the other hand, the main drawbacks of impedance sensors seem to be related to the skin impedance that limits the depth at which it is possible to detect the occurrence of extravasation. Consequently, the capability of measuring extravasation strongly depends on the location at which the event occurs, while a more robust detection system within the vein is desirable. We also found out that the impedance-based sensor presents a different sensitivity depending on the injection rate. In particular, it appears to be more difficult to detect extravasation events in the case of slower flow rates. Anyhow, one should consider that the impedance sensors presented here are mainly noninvasive patches: the presence of an impedance sensor directly inside the vein could be of help in improving the dependence of the detection on the location of the extravasation event and on the fluid injection rate. Furthermore, especially in the case of impedance sensors, we think it could be of notable importance to be able to measure a resistance or impedance “baseline” just before starting the infusion. This aspect would help determine a patient-specific threshold of bioelectrical characteristics according to the patient’s skin.

Overall, due to the advantages that all these platforms show separately, we think multimode detection technologies—i.e., devices that combine different single-sensors modalities—could be of interest in achieving ever better performance. We presented in this review some examples of such devices. However, it is necessary to conduct more clinical trials related to this field, since, among the articles reported here, the sensitive platforms were only tested on animals or artificial skin models.

Some other important features emerged from the reviewed articles, which should be taken into account in the design of extravasation detection systems. First, they should be easy for doctors and nurses to handle. In addition, it is critically significant to consider a lightweight, small-size technology to be employed in pediatric cases. It would be also ideal to have a low-power embedded processing system to localize the catheterization site in order to monitor the procedure for a long time without hindering patients’ mobility and capable of alerting physicians if extravasation is detected.

In conclusion, there are several patch-based devices in the scientific literature as well as commercially available products. In particular, most of the transduction technologies have still been demonstrated on patch implementation, including multiparameter approaches, with several examples reported in the main text. On the other hand, we think that a sensorized catheter-like device can offer several advantages for the local measurement compared to the more standard patch-based devices. This kind of technology could be more sensitive to extravasation events (i.e., detecting small volumes) since it is localized inside the tissue, giving also the possibility to monitor the position of the catheter with respect to the vessel walls. Such a device could also shorten the overall workflow since the catheter and cannula are yet present as they are part of the medical procedure. Hence, no additional external devices are needed, allowing for less equipment and easier setup. Furthermore, if the fabrication process of the electrodes to be embedded on the catheter is optimized, it could result in a less expensive device with respect to patch solutions.
